# Association of paternal age at birth and the risk of breast cancer in offspring: a case control study

**DOI:** 10.1186/1471-2407-5-143

**Published:** 2005-10-31

**Authors:** Ji-Yeob Choi, Kyoung-Mu Lee, Sue Kyung Park, Dong-Young Noh, Sei-Hyun Ahn, Keun-Young Yoo, Daehee Kang

**Affiliations:** 1 Department of Preventive Medicine, Seoul National University College of Medicine, 28 Yongon-Dong Chongno-Gu, Seoul 110-799 Korea; 2 Department of General Surgery, Seoul National University College of Medicine, 28 Yongon-Dong Chongno-Gu, Seoul 110-799 Korea; 3 Department of General Surgery, Ulsan University College of Medicine, 388-1 Pungnap-2dong Songpa-gu, Seoul 138-736, Korea; 4 Cancer Research Institute, Seoul National University College of Medicine, 28 Yongon-Dong Chongno-Gu, Seoul 110-799 Korea

## Abstract

**Background:**

Older paternal age may increase the germ cell mutation rate in the offspring. Maternal age may also mediate *in utero *exposure to pregnancy hormones in the offspring. To evaluate the association between paternal and maternal age at birth with the risk of breast cancer in female offspring, a case-control study was conducted in Korea.

**Methods:**

Histologically confirmed breast cancer cases (n = 1,011) and controls (n = 1,011) with no present or previous history of cancer, matched on year of birth and menopausal status, were selected from several teaching hospitals and community in Seoul during 1995–2003. Information on paternal and maternal ages and other factors was collected by interviewed questionnaire. Odds ratio (OR) and 95% confidence interval (95% CI) were estimated by unconditional logistic regression model adjusting for family history of breast cancer in 1^st ^or 2^nd ^degree relatives, and lifetime estrogen exposure duration.

**Results:**

The risk of breast cancer significantly increased as the paternal age increased (p for trend = 0.025). The association was stronger after controlling for maternal age; women whose fathers were aged ≥40 years at their birth had 1.6-fold increased risk of breast cancer compared with fathers aged <30 years. This association was profound in breast cancer cases in premenopausal women (OR = 1.9, 95% CI = 1.12–3.26, for paternal aged ≥40 vs. <30) (p for trend = 0.031). Although the risk of breast cancer increased as maternal age increased up to the intermediate, and then reduced; the risks in women whose mother were aged 25–29, 30–34, and ≥35 yrs at birth compared to women whose mothers were aged <25 years, were 1.2, 1.4, and 0.8, respectively, the trend was not significant (p for trend = 0.998).

**Conclusion:**

These findings suggest that older paternal age increases the risk of breast cancer in their female offspring.

## Background

There have been growing evidences that prenatal factors may play an important role in determining breast cancer risk in adult life. These factors are hypothesized to affect breast cancer risk by altering the hormonal environment of the developing fetus [1], or by affecting the cumulative frequency of germ cell mutations [2,3].

A numbers of epidemiologic research have suggested that older parental ages may influence risk for subsequent development of breast cancer later in their lives. Several studies found a slightly increased risk of breast cancer for women whose mothers were older [4-11], but not all [12-25]. Similarly, there have been conflicting results with regard to breast cancer risk according to paternal age [4,5,8,9,13,16,18,20,24,25]. Recently, Innes et al [7] suggested that there was a positive trend in risk of breast cancer with increasing paternal age in young women.

These inconsistent results from previous studies might be due to several factors; small numbers of breast cancer cases, subject selection (e.g., age at diagnosis, different ethnicity), improper adjustment of covariates and paternal and maternal ages mutually, other study design issue (no information on known risk factors).

There is a trend toward higher paternal and maternal ages predominantly in Korea as well as in developed countries [26]. To our knowledge no studies published to date have specifically addressed the association between paternal and maternal ages and breast cancer risk in Asian women. We evaluate the independent effect of both paternal and maternal ages at birth on the risk of breast cancer of their daughters in a large case-control study in Korean.

## Methods

The cases consisted of a consecutive series of breast cancer patients admitted to three teaching hospitals located in Seoul, Korea (SNUH, Borame, and Asan) between 1995 and 2003. The control subjects consisted of non-cancer patients admitted to the same hospitals as the cases in the same period and of healthy women who participated in the community health screening program provided by a teaching hospital located in Seoul (EWUMC) in 2003. The study design was approved by the Institutional Review Board of Seoul National University Hospital, and the subjects provided their informed consents prior to participation in the study.

Of 1,999 histologically confirmed incident breast cancer patients and 1,548 cancer-free controls, 1,709 breast cancer cases and 1,412 cancer-free controls were eligible after exclusion of subjects with previous history of cancer or previous history of hysterectomy and/or oophorectomy due to cervical, ovarian cancer or its precursors. The control group consisted of 577 healthy women and of 835 hospital controls with non-cancerous diseases including infection or stone of gall bladder/bile duct (26%), benign breast disease (e.g. fibroadenomas, fibrocystic disease, mastitis, etc.) (17%), acute appendicitis (14%), hemorrhoid (8%), hernia/perforation (7%), lipoma (2%), and the others (26% included liver injury, cellulitis, chronic bowl disease, benign vascular disease, ulcer, etc.). The proportion of atypical hyperplasia was estimated less than 0.2% among benign breast disease in Korean [27].

Information on demographic characteristics, current and previous residence, education, marital status, family history of breast cancer in the 1^st ^and 2^nd ^degree relatives, reproductive and menstrual factors, life-style habits including cigarette smoking, alcohol consumption, oral contraceptive use, and hormone replacement therapy was collected by trained interviewers using a structured questionnaire.

After exclusion of subjects with missing value of either maternal or paternal age (n = 185 in cases; n = 320 in controls), cases were frequency matched to controls by 10-year of birth group (before 1930, 1930–1939, 1940–1949, 1950–1959, 1960–1969, after 1970) and menopausal status. The final study population consisted of 1,011 cases and 1,011 controls. The distribution of matched controls was similar to that of unmatched controls (missing either paternal or maternal age) with regard to age, family history of breast cancer in 1^st ^or 2^nd ^degree relatives, and lifetime estrogen exposure duration. Risk factors profiles were not different between hospital and healthy community controls [28]; although community controls were older than hospital controls, the distributions of most known risk factors (e.g., education, family history of breast cancer in 1^st ^and 2^nd ^degree relatives) were similar. The means of paternal and maternal ages at birth were not significantly different between hospital and community controls (32.7 vs. 32.1 in paternal age, p = 0.222; 28.3 vs. 28.4 in maternal age, p = 0.807 examined by *t*-test). The means of paternal and maternal age at birth of patients with benign breast disease among controls were also different from neither hospital controls nor overall controls (data not shown). Although eighty-seven percent of hospital controls and 97% of community controls came from the same catchment areas (Seoul and suburbs), other demographical characteristics (e.g., age, paternal age, maternal age) were similar between hospital and community controls. Thus, the final statistical analyses were done by adjusting for all significant covariates identified from the initial analysis.

The associations between factors of interest and breast cancer risk were estimated as odds ratios (ORs) and 95% confidence intervals (CIs) by unconditional logistic regression model adjusting for family history of breast cancer in 1^st ^or 2^nd ^degree relatives (yes/no), and lifetime estrogen exposure duration (yrs) (presenting the number of years of exposure to menstrual cycles, which is calculated according to the age at menarche and age at interview for premenopausal women and age at menarche and age at menopause for postmenopausal women), which were identified as the significant covariates (p value < 0.05) in the initial analysis. The variables included in the logistic model were selected among age (yrs), education (under or at middle school, at high school, at or over college), family history of breast cancer in 1^st ^or 2^nd ^degree relatives (yes/no), lifetime estrogen exposure duration (yrs), age at full-term pregnancy or nulliparous (<25 yrs, 25–29 yrs, ≥30 yrs or nulliparity), cigarette smoking (smoked at least 400 cigarettes/lifetime, yes/no), frequency of alcohol consumption (<1/month, 1–3/month, ≥1/week) and body mass index (BMI) (<25.0 kg/m^2^, 25.0–30.0 kg/m^2^, ≥30.0 kg/m^2^). Only the adjusted estimates were reported in the results because the change in the β coefficient for any level of the paternal and maternal ages relative to the referent was less than 20% between unadjusted estimates and those adjusted for family history of breast cancer in 1^st ^or 2^nd ^degree relatives (yes/no), and lifetime estrogen exposure duration (yrs). Tests for trend in risk were conducted by treating categorical values as a continuous variable.

Paternal and maternal ages of subjects at birth were first compared using *t*-test and the estimates of odds ratios (ORs) with adjustment for other covariates were obtained using unconditional logistic regression analysis. We classified subjects into four groups according to paternal age at birth (<30, 30–34, 35–39, ≥40) and maternal age at birth (<25, 25–29, 30–34, ≥35). Separate analysis was conducted with and without the other paternal or maternal age mutually adjusting. To evaluate the independent effect of paternal and maternal ages, the ORs were calculated after stratified by the each paternal and maternal age group. Finally, we assessed the association of paternal and maternal ages at birth with breast cancer after stratified by menopausal status. All statistical analyses were performed using STATA version 8.0 (Stata corporation, College Station, TX).

## Results

Family history of breast cancer in 1^st ^and 2^nd ^degree relatives (OR = 2.2, 95% CI = 1.47–3.38), lifetime estrogen exposure duration (per 10 years) (OR = 1.2, 95% CI = 1.05–1.41), ≥30 age at first full-term pregnancy or nulliparity (OR = 1.3, 95% CI = 1.04–1.76) and BMI ≥30 kg/m^2 ^(OR = 2.0, 95% CI = 1.07–3.78) increased the risk of breast cancer significantly after adjusting for family history of breast cancer in 1^st ^and 2^nd ^degree relatives, and lifetime estrogen exposure duration (Table 1).

The mean of paternal age at birth was significantly different between cases and controls (33.1 yrs vs. 32.5 yrs; OR = 1.1, 95% CI = 1.00–1.28 per 10 yrs), however, the mean of maternal age was not (28.7 yrs vs. 28.3 yrs; OR = 1.1, 95% CI = 0.97–1.28 per 10 yrs) (Table 2). The risk of breast cancer showed a significantly increased as paternal age at birth increased (p for trend = 0.025). The association of paternal age with the risk of breast cancer was pronounced after controlling for maternal age; women whose fathers were aged 30–34, 35–39, and ≥40 yrs at their births, had 1.0, 1.0, and 1.6-fold increased risk of breast cancer compared with women whose fathers were aged <30 years, respectively. The risk of breast cancer increased as maternal age increased up to the intermediate, and then reduced; the risks in women whose mother were aged 25–29, 30–34, and ≥35 yrs at birth compared to women whose mothers were aged <25 years, were 1.2, 1.4, and 0.8, respectively (Table 2). The most remarkable risk of breast cancer was observed for women with higher paternal age (≥40 yrs) and intermediate maternal age (30–34 yrs) compared to women with lowest paternal (<30 yrs) and maternal ages (<25 yrs) (OR = 2.8, 95% CI = 1.74–4.62) (Table 3).

When the association was evaluated after stratified by menopausal status, the association of paternal age at birth in breast cancer was stronger in premenopausal women. Women whose fathers were aged 30–34, 35–39 and ≥40 years at their birth had 1.1, 1.2 and 1.9-fold increased risk of breast cancer compared with women whose fathers were aged <30 years, respectively (p for trend = 0.031). In contrast with paternal age, there was no significant trend between maternal age at birth and risk of breast cancer in premenopausal women (p for trend = 0.361) (Table 4).

## Discussion

The results of the present study suggested that older paternal age at the time of a child's birth was associated with an increased risk of breast cancer in female offspring, which was enhanced after controlling maternal age. The effect appears to be stronger in premenopausal women. The results also suggested that there was no consistent pattern of association between maternal age at birth and risk of breast cancer although the risk increased up to women with mothers aged 30–34 years old and then reduced in women with maternal age older than 35 years old.

The mean age at first full-term pregnancy was 22.0 years old in marriage cohort before 1980 while 27.3 years old in the cohort of 2000–2003 in Korea [29], which shows that the age at first full-term pregnancy became progressively older in younger age groups. This study also found that the paternal and maternal age were significantly higher in the subjects born after 1950 than in those before 1950 for both case and control groups.

The results of previous studies of the relationships of the risk of breast cancer with paternal and maternal ages are summarized in Table 5 and these are not consistent. Four studies indicating that there was a increasing trend of the risk of breast cancer in women having older father, were consisted with the present study [5,7-9]. A number of previous studies found no statistically significant association between paternal age and the risk of breast cancer in population-based large scale studies. Most of negative studies did not adjust known risk factors for breast cancer (i. e., family history of breast cancer in 1^st ^and 2^nd ^degree relatives and reproductive factors). In contrast, Hodgson et al [5] showed that there was a positive association between breast cancer and paternal age in African-American only. Innes et al [7] and Le Marchand et al [9] also reported a significant linear trend in breast caner risk with paternal age only in young cancer cases.

Most previous studies showed that maternal age was not associated with breast cancer risk in white women. However, the finding of this study that the breast cancer risk increased with increasing maternal age up to the intermediate (30–34 years old), then reduced for older than 35 years old, is consistent with previous epidemiological studies [5,11-13,24,30]. Experimental evidences also support the association between maternal age and estrogen level during pregnancy [31-33]. Panagiotopoulou et al [32] showed the inverse U-shaped relationship of maternal age to estrogen levels; there was a peak of estrogen levels in the intermediate then there was a reduction in estrogen levels with maternal age. This pattern that maternal age was not linear relationship with estradiol level during pregnancy and/or generally not in a dose-dependent gradient with age was observed among other recent studies [31,33]. These results supported that subjects whose mothers were oldest at the time of birth were not in the highest risk group in this study and it may be biologically relevant because there is a perimenopausal reduction in estrogens.

With regard to menopausal status, most previous results between the risk of breast cancer and maternal age including ours are inconsistent [6,7,9,22]. Potential reasons for inconsistent association between maternal age and breast cancer risk include differences in other maternal factors (i.e., maternal diet, pregnancy complication, and maternal reproductive history), covariates adjusted, or ethnicity.

Data published so far shows that maternal and paternal ageing may affect offspring by different mechanisms. Higher concentration of estrogen *in utero *could create a fertile soil for cancer initiation with regard to maternal age [1]. There is some evidence that high prenatal estrogen level affects the morphology of the mammary gland (i.e., the number of ductal branching, the density of terminal end buds). An increased number of epithelial and stromal cells offer more targets for carcinogens and greater probability for genetic/epigenetic events that affect the susceptibility of the breast cancer [34]. Higher paternal age has been implicated to be responsible for increases in chromosomal aberrations and genetic disorders [35], of which the risk increases with paternal age, but maternal age has little or no effect on the risk after controlling for paternal age [3]. Spermatogomia undergo continuous cell divisions throughout the lifetime of the adult male, while oocytes undergo only one cell division between puberty and fertilization [2,3]. This difference may provide a greater opportunity for germ cells of the father to experience errors of DNA replication in regard to delayed parenthood. Furthermore, the ability to respond to mutagens with germ-cell apoptosis in order to avoid genetically altered spermatozoa decreased with paternal age [36], while oocytes have an efficient DNA repair system which is independent of maternal age [37].

Although maternal and paternal ages were correlated, we were able to ascribe the risk of breast cancer to the paternal age independent on maternal age. There are, however, several issues in the present results to consider whether the observed effects can be due to confounding or bias. First, potential selection bias because offspring with higher socioeconomic status may have detected their breast cancer earlier than those with lower socioeconomic status and because individuals with higher socioeconomic status may tend to have children later. In this study, however, the education levels of study subjects (offspring) were not associated with paternal or maternal ages and education levels did not change the β coefficient of paternal and maternal ages less than 15%, thus education levels were not included in the final model. Moreover, controlling for subjects' education levels in narrower categories (5 categories; at or under elementary school, at middle school, at high school, at college, and at graduate school) did not significantly alter the breast cancer risk of paternal and maternal age. However, we could not adjust parental education since we had no information about parental education levels. Seventy-nine percent of cases and 91% of controls came from the same catchment areas (Seoul and suburbs). However, it did not indicate that the subjects came from the different study bases since the hospitals participated in the study were university hospitals which covered the patients in the whole country according to the current health delivery system in Korea [38]. Even if the catchment areas were different between cases and controls, this difference may push the association toward the null since more controls came from urban area where subjects had higher education levels which was associated with increased breast cancer risk. Second, possible unadjusting confounders from other risk factors for breast cancer in adult life [39,40], but the risk of paternal and maternal ages on breast cancer remained same even after adjusting the established risk factors for breast cancer. However, other studies have mostly not found similar results and since small relative risks are involved and chance remains a likely explanation, the results of this moderately sized case-control study should be interpreted with extreme cautions.

## Conclusion

This is the first and largest report about paternal and maternal ages and breast cancer in Asian women. These findings suggest that paternal age is associated with an increased risk of breast cancer in female offspring. However, further prospective study is needed to verify the present findings.

## Competing interests

The author(s) declare that they have no competing interests.

## Authors' contributions

J-YC conceived of the study, conducted data analysis, and drafted the manuscript. K-ML contributed to the design and management of data. SKP participated in data analysis and interpretation. D-YN, S-HA, and K-YY designed the study. DK conceived of and designed the study, obtaining funding and drafted the manuscript. All authors read and approved the final manuscript.

## Pre-publication history

The pre-publication history for this paper can be accessed here:



## Figures and Tables

**Table 1:** Selected characteristics for 1,011 breast cancer cases and 1,011 controls matched by year of birth and menopausal status

**Risk factor**	**Cases (%)**	**Control (%)**	**OR (95% CI)**
		
	n = 1,011	n = 1,011	
**Age (yrs) (mean ± SD)**	47.7 ± 10.9	47.7 ± 11.7	p = 0.984*
**Education**			
Under or at middle school	315 (31.3)	319 (31.7)	1.0
At high school	378 (37.5)	407 (40.5)	1.0 (0.80–1.24)
At or over college	315 (31.3)	280 (27.8)	1.2 (0.98–1.57)
**FHBC^‡^**			
No	937 (92.7)	976 (96.5)	1.0
Yes	74 (7.3)	35 (3.5)	2.2 (1.47–3.38)
**Age at FFTP^‡ ^(yrs) or nulliparity**			
<25	325 (32.2)	349 (34.9)	1.0
25–29	477 (47.3)	471 (47.1)	1.1 (0.92–1.38)
≥30 or nulliparity	206 (20.4)	181 (18.1)	1.3 (1.04–1.76)
P for trend			p = 0.027
**LEE^‡ ^(yrs) (mean ± SD)**	29.1 ± 6.9	28.3 ± 7.4	1.2 (1.05–1.41)^†^
**BMI (kg/m^2^)**			
<25.0	748 (74.4)	769 (76.8)	1.0
25.0–29.9	227 (22.6)	216 (21.6)	1.0 (0.84–1.29)
≥30.0	31 (3.1)	16 (1.6)	2.0 (1.07–3.78)
**Smoking status**			
Nonsmoker	933 (92.4)	943 (93.4)	1.0
Smoker	77 (7.6)	67 (6.6)	1.2 (0.85–1.70)
**Alcohol drinking**			
<1/month	756 (74.8)	709 (72.3)	1.0
1–3/month	178 (17.6)	211 (21.5)	0.8 (0.64–1.02)
≥1/week	77 (7.6)	61 (6.2)	1.2 (0.86–1.76)

OR of case vs. matched control adjusted for family history of breast cancer in 1^st ^or 2^nd ^degree relatives and lifetime estrogen exposure duration

*P value by student t test, ^†^OR per 10 year, ^‡^FHBC, Family history of breast cancer in 1^st ^or 2^nd ^degree relatives; FFTP, first full-term pregnancy; LEE, lifetime estrogen exposure duration

**Table 2:** Association between paternal and maternal ages at birth and risk of breast cancer in daughters

**Age at birth of the subjects (yrs)**	**Cases (%)**	**Controls (%)**	**OR (95% CI)^1^**	**OR (95% CI)^2^**
**Paternal age**				
Mean ± SD	33.1 ± 7.5	32.5 ± 7.2	1.1 (1.00–1.28)^†^	
<30	362 (35.8)	400 (39.6)	1.0	1.0
30–34	266 (26.3)	261 (25.8)	1.2 (0.92–1.45)	1.0 (0.79–1.34)
35–39	169 (16.7)	174 (17.2)	1.1 (0.83–1.40)	1.0 (0.71–1.39)
≥40	214 (21.2)	176 (17.4)	1.4 (1.07–1.76)	1.6 (1.04–2.32)
P for trend			0.025	0.090
**Maternal age**				
Mean ± SD	28.7 ± 6.4	28.3 ± 6.5	1.1 (0.97–1.28)^†^	
<25	285 (28.2)	330 (32.6)	1.0	1.0
25–29	302 (29.9)	297 (29.4)	1.2 (0.97–1.54)	1.2 (0.95–1.56)
30–34	237 (23.4)	192 (19.0)	1.5 (1.18–1.95)	1.4 (1.00–1.94)
≥35	187 (18.5)	192 (19.0)	1.1 (0.88–1.48)	0.8 (0.55–1.27)
P for trend			0.079	0.998

^1^OR adjusted for family history of breast cancer in 1^st ^or 2^nd ^degree relatives and lifetime estrogen exposure duration

^2^OR adjusted for maternal age or paternal age (mutual parental age, categorical value) in addition to the variables in the model 1

^†^OR per 10 year

**Table 3:** OR (95% CI) of combined effect of paternal age and maternal ages on risk of breast cancer [cases/controls]

**Paternal age (yrs)**	**Maternal age (yrs)**			
	
	**<25**	**25–29**	**30–34**	**≥35**
**<30**	1.0 [230/273]	1.3 (0.97–1.81) [122/116]	1.1 (0.45–2.86) [9/10]	1.1 (0.07–17.69) [1/1]
**30–34**	1.2 (0.76–1.87) [46/47]	1.3 (0.96–1.76) [132/127]	1.3 (0.91–1.89) [81/79]	1.1 (0.38–2.97) [7/8]
**35–39**	1.6 (0.55–4.81) [8/6]	1.1 (0.67–1.70) [41/46]	1.4 (1.00–2.07) [83/74]	0.9 (0.55–1.42) [37/48]
**≥40**	0.3 (0.04–3.07) [1/4]	1.2 (0.43–3.60) [7/8]	2.8 (1.74–4.62) [64/29]	1.3 (0.96–1.75) [142/135]

OR adjusted for family history of breast cancer in 1^st ^or 2^nd ^degree relatives and lifetime estrogen exposure duration

**Table 4:** Association between parental ages at birth and risk of breast cancer in daughters after stratified by menopause status

**Age at birth of the subjects (yrs)**	**Cases (%)**	**Controls (%)**	**OR (95% CI)^1^**	**OR (95% CI)^2^**
**Premenopause**				
**Paternal age**				
Mean ± SD	33.7 ± 7.0	32.9 ± 6.6	1.16 (0.98–1.38)^†^	
<30	183 (31.6)	210 (36.3)	1.0	1.0
30–34	162 (28.0)	161 (27.8)	1.1 (0.85–1.54)	1.1 (0.76–1.50)
35–39	108 (18.7)	105 (18.1)	1.1 (0.82–1.60)	1.2 (0.77–1.85)
≥40	126 (21.8)	103 (17.8)	1.4 (1.00–1.93)	1.9 (1.12–3.26)
P for trend			0.062	0.031
**Maternal age**				
Mean ± SD	29.1 ± 5.8	28.8 ± 6.1	1.06 (0.87–1.29)^†^	
<25	133 (23.0)	159 (27.5)	1.0	1.0
25–29	194 (33.5)	187 (32.3)	1.3 (0.93–1.73)	1.2 (0.86–1.70)
30–34	147 (25.4)	120 (20.7)	1.5 (1.06–2.07)	1.2 (0.78–1.88)
≥35	105 (18.1)	113 (19.5)	1.1 (0.76–1.55)	0.7 (0.38–1.15)
P for trend			0.363	0.361
**Postmenopause**				
**Paternal age**				
Mean ± SD	32.2 ± 8.1	31.8 ± 8.0	1.11 (0.94–1.32)^†^	
<30	179 (41.4)	190 (44.0)	1.0	1.0
30–34	104 (24.1)	100 (23.2)	1.2 (0.82–1.65)	1.0 (0.66–1.48)
35–39	61 (14.1)	69 (16.0)	1.0 (0.65–1.49)	0.8 (0.47–1.35)
≥40	88 (20.4)	73 (16.9)	1.4 (0.94–2.03)	1.2 (0.66–2.29)
P for trend			0.173	0.804
**Maternal age**				
Mean ± SD	28.2 ± 7.1	27.7 ± 6.9	1.16 (0.95–1.41)^†^	
<25	152 (35.2)	171 (39.6)	1.0	1.0
25–29	108 (25.0)	110 (25.5)	1.1 (0.80–1.60)	1.2 (0.80–2.29)
30–34	90 (20.8)	72 (16.7)	1.6 (1.05–2.31)	1.6 (0.97–2.69)
≥35	82 (19.0)	79 (18.3)	1.2 (0.82–1.78)	1.1 (0.58–2.03)
P for trend			0.112	0.415

^1^OR adjusted for family history of breast cancer in 1^st ^or 2^nd ^degree relatives and lifetime estrogen exposure duration

^2^OR adjusted for maternal age or paternal age (mutual parental age, categorical value) in addition to the variables in the model 1

^†^OR per 10 year

**Table 5:** Summary of results from previous studies of parental ages and breast cancer development

**Study**	**Country**	**N (# cases)**	**Age (yrs)**	**Paternal age (PA)**	**RR**	**Maternal age (MA)**	**RR**	**Adjusting**		**Restricted**
									
								Mutually*	**Covariates**^†^	
**Cohort study**										
Colditz et al, 1991	US	118,309 (1,976)	3055	≥39 vs. <20	0.9 (0.56–1.45)	≥39 vs. <20	0.9 (0.57–1.39)	YES	YES	-
Zhang et al, 1995	US	2,662 (149)	29–62	≥36 vs. <29	0.8 (0.5–1.4)	26–31 vs. <26	1.5 (1.0–2.4)	-	YES	-
Holmberg et al^1^, 1995	US	384,769 (1,967)	-	≥45 vs. <20	0.8 (0.48–1.28)	≥45 vs. <20	1.3 (0.85–1.98)	-	YES	-
Hemminki et al, 1999	Sweden	3,800,000 (8,877)	15–53	40–49 vs. <25	1.1 (0.94–1.18)	40–49 vs. <20	1.1 (0.91–1.27)	YES	-	PA in sporadic cases
Hilakivi-Clarke et al, 2001	Finland	3,447 (177)	-	-	-	-	NS	-	-	-

Study	Country	Cases/controls	Age (yrs)	Paternal age (PA)	OR	Maternal age (MA)	OR	Adjusting		Remark
									
								Mutually*	Covariates^†^	

**Case-control study**										
Standfast et al, 1967	US	229/229	40–44	-	-	29.1 vs. 28.2	^2^p < 0.05	-	-	-
Henderson et al, 1974	US	308/308	<64	31.5 vs. 30.7	NS	27.3 vs. 26.3	P < 0.01	-	-	-
Rothman et al, 1980	International	4339/12760	-	-	-	35–39 vs. <20	1.30	-	YES	-
Baron et al, 1984	UK	971/971	≤50	-	-	21–25 vs. ≤20	1.4 (0.92–2.18)	-	YES	-
Le Marchand et al, 1988	US	153/461	<45	36–59 vs. 19–26	1.4 (0.81–2.41)	30–46 vs. 23–26	1.7 (0.99–2.78)	-	-	Pts <33 yrs
Janerich et al, 1989	US	801/2647	-	Per 10-yrs	1.2 (1.07–1.33)	Each 10-yrs	1.2 (1.09–1.41)	-	-	-
Thompson et al, 1990	US	2492/2687	20–54	-	-	35–39 vs. <20	1.5 (1.10–1.93)	-	YES	parous
Hsieh et al, 1991	International	927/2616	≥35	-	-	Each 5-yrs	1.1 (1.01–1.10)	-	YES	postmenopause
Ekbom et al, 1992	Sweden	458/1197	≥35	-	-	Each 5-yrs	1.0 (0.92–1.12)	-	-	-
Janerich et al, 1994	US	2414/9138	-	≥45 vs. <25	1.0 (0.76–1.28)	≥40 vs. <20	1.1 (0.87–1.37)	-	YES	-
Sanderson et al, 1995	US	1147/1399	21–45, 50–64	-	-	≥35 vs. <25	1.0 (0.7–1.4), 1.0 (0.7–1.5)	-	-	-
Ekbom et al, 1997	Sweden	1068/2727	-	-	-	Each 5-yrs	1.1 (0.99–1.14)	-	-	-
Newcomb et al, 1997	US	1253/1121	-	≥40 vs. ≤24	0.9 (0.68–1.24)	≥40 vs. ≤20	0.9 (0.62–1.37)	-	YES	-
Weiss et al, 1997	US	2173/1990	20–55	-	-	≥35 vs. <20	0.9 (0.7–1.3)	-	YES	-
Innes et al, 2000	US	484/2870	14–37	≥40 vs. 25–29	1.5 (1.03–2.23)	≥35 vs. 20–24	1.9 (1.18–3.18)	YES	-	-
Titus-Ernstoff et al, 2002	US	5629/5928	50–79	-	-	≥40 vs. 25–29	1.3 (0.90–1.79)	-	-	postmenopause
Mellemkjǽr et al, 2003	Denmark	881/3423	<40	-	-	≥30 vs. <25	1.1 (0.90–1.36)	-	-	-
Hodgson et al, 2004	US	280/236	18–74	35–56 vs. 23–27	1.5 (0.7–3.2)	≥23–27 vs. 19–22	3.5 (2.0–5.9)	YES	-	PA in African American

* Adjusted for mutually parental age; ^†^Adjusted for other risk factors of breast cancer risk including family history of breast cancer in 1^st ^or 2^nd ^degree relatives and reproductive history; ^1^Mortality of breast cancer, relative hazard; ^2^Paired *t *test
